# Prevalence of hospital‐acquired malnutrition and modifiable determinants of nutritional deterioration during inpatient admissions: A systematic review of the evidence

**DOI:** 10.1111/jhn.13009

**Published:** 2022-04-26

**Authors:** Alyssa R. Cass, Karen E. Charlton

**Affiliations:** ^1^ School of Medicine, Faculty of Science, Medicine and Health University of Wollongong Wollongong NSW Australia; ^2^ Illawarra Health & Medical Research Institute Wollongong NSW Australia

**Keywords:** inpatients, malnutrition, nutrition assessment, nutritional status

## Abstract

**Background:**

Malnutrition affects between 20% and 50% of hospital inpatients on admission, with further declines expected during hospitalisation. This review summarises the existing literature on hospital‐acquired malnutrition that examines the magnitude of nutritional deterioration amongst adult inpatients and identifies preventable barriers to optimising nutrition support during episodes of care.

**Methods:**

A systematic review was conducted to answer the question: Among adult hospital inpatients, the presence of which modifiable factors contribute to hospital‐acquired malnutrition? A database search was conducted between the 24 April and 30 June 2020 using CINAHL, MEDLINE, Scopus and PubMed databases according to a protocol registered with PROSPERO (CD42020182728). In addition, issues of the 10 top clinical nutrition journals published during the period of from 1 April 2015 to 30 March 2020 were hand‐searched.

**Results:**

Fifteen articles were eligible for inclusion from a total of 5944 retrieved abstracts. A narrative synthesis of evidence was completed because of the high level of heterogeneity in methodologies. Nutritional deterioration is common among previously well‐nourished and nutritionally compromised patients, with studies reporting that 10%–65% of patients experienced nutritional decline. Frequently reported barriers were mealtime interruptions, meal dissatisfaction, procedure‐related fasting, effects of illness or treatment, chewing difficulties, poor appetite and malnutrition as a low clinical priority.

**Conclusions:**

The findings of this review support the need for routine nutritional risk screening throughout each hospital admission with hospital‐acquired malnutrition affecting up to 65% of inpatients. Clear establishment of the roles and responsibilities of each member within multidisciplinary healthcare teams in the provision of nutrition care and cost–benefit analyses are recommended to demonstrate the effectiveness of changes to models of care.

## Key points

- Among adult hospital inpatients, the presence of which modifiable factors contribute to hospital‐acquired malnutrition?
- Nutritional deterioration was identified in 10%–65%of patients from 15 eligible studies, with barriers to nutritional adequacy frequently reported on both the institution and patient levels.
- There is a need for routine nutritional risk screening throughout each hospital admission, shared responsibility for nutrition care across the broader multidisciplinary healthcare team, and greater emphasis to be placed on patient satisfaction with hospital meal services.

## Graphical abstract

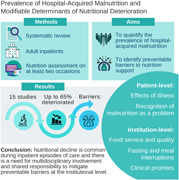

## INTRODUCTION

Malnutrition affects between 20% and 50% of hospital inpatients.^1^, ^2^, ^3^ If untreated, a further two‐thirds of patients admitted with malnutrition will experience a decline during the course of their admission, whereas one‐third of well‐nourished patients may become malnourished.^4^ Malnutrition in hospital may be precipitated by iatrogenic factors, barriers to intake and complex physiological and metabolic alterations accompanying the acute inflammatory response that disrupt normal nutrient utilisation and promote catabolism and/or hypermetabolism.^5^, ^6^, ^7^ In some cases of disease‐related malnutrition, nutrition support alone may be inadequate to prevent further nutritional decline despite energy provision corresponding with measured energy expenditures.^5^, ^8^

Hospital malnutrition is a predictor of increased length of stay, impaired wound healing, increased risk of infections and complications, and increased morbidity and mortality.^1^, ^4^ Thus, malnourished patients have more substantial care needs with a greater reliance on hospital resources resulting in higher healthcare costs.^1^, ^3^, ^4^, ^6^, ^7^, ^8^, ^9^, ^10^, ^11^, ^12^, ^13^ This has led to the integration of nutrition screening into hospital admission protocols. However, whether nutrition screening is required to be undertaken at all, as well as the timeframe in which it is to be completed, is at the discretion of the regional healthcare governing body. In New South Wales (NSW), nutrition screening using a validated tool is a requirement for all hospitals. It is recommended to be undertaken within the first 24 h and weekly thereafter during an acute admission or following a change in a patient's clinical condition.^14^ Despite this, routine nutrition screening using validated tools is not always performed and, often, nutrition intervention is more reactive rather than proactive.^15^ Ongoing nutrition screens may be disregarded because of competing clinical priorities, which remains a primary challenge to circumventing persistently high rates of hospital‐acquired malnutrition.^16^ Although malnutrition in hospitalised patients has been thoroughly studied in the past, the evidence examining the magnitude of malnutrition acquired during hospital stays, and institution‐level factors that contribute to worsening of nutritional status during admission is less concrete. Taking a proactive approach to combatting hospital‐acquired malnutrition necessitates that system‐level barriers are clearly identified to develop targeted solutions.

Documentation in medical records does not typically delineate cases of hospital‐acquired malnutrition from cases of community‐acquired malnutrition, in which patients present with pre‐existing malnutrition on admission. The former can be further categorised based on preventable and non‐preventable aetiologies. Preventable hospital‐acquired malnutrition may or may not be accompanied by injury or inflammation with consequent increases in nutritional requirements, where nutritional requirements have not been met. Non‐preventable hospital‐acquired malnutrition refers to malnutrition in the presence of injury or inflammation, where nutritional status remains compromised despite adequate nutritional intake.^8^ The Australian Commission on Safety and Quality in Health Care recognises malnutrition as one of 16 Hospital‐Acquired Complications (HACs), defined as nosocomial conditions for which the clinical risk may be mitigated through appropriate preventive strategies. In July 2018, a new Risk Adjustment Model was implemented by the Independent Hospital Pricing Authority (IHPA). Under this model, hospitals receive financial penalties to reimbursements when HACs, including hospital‐acquired malnutrition, are coded.^17^ The financial burden associated with caring for patients with hospital‐acquired malnutrition and the additional onus now placed on Australian hospitals under the IHPA model creates strong incentive to identify and address causes of preventable hospital‐acquired malnutrition.

Studies examining modifiable determinants of hospital‐acquired malnutrition are limited. In one study, 76% of malnourished patients experienced at least one institution‐level care gap including poor dietitian–physician communication, inappropriate nil‐by‐mouth (NBM) orders or inaccurate dietetic discharge instructions.^18^ NBM is often prescribed inappropriately as a result of updated clinical practice guidelines not being widely adopted.^19^, ^20^ Evidence suggests a strong association between any care‐related gap and increased length of hospital stay.^19^, ^20^, ^21^, ^22^, ^23^ However, the generalisability of these studies is limited because of a reliance on single‐centre data and having been conducted prior to full implementation of the pricing model. Preventable components may also extend beyond these predetermined classifications, and more precisely identifying these shortcomings will enable the development of protocols to mitigate preventable causes.

Recommendations have been made for the establishment of targeted interventions addressing barriers related to food service, mealtime and nutrition care, which include the need for a multidisciplinary team approach and institutional culture that prioritises nutrition more broadly within the context of clinical care. However, specific nutrition care responsibilities of each member of the team have yet to be established.^15^ Identifying existing shortcomings is a critical first step to promoting changes in practice through evidence‐based education of administrators on the direct benefit of enhanced food service and care‐related processes to patient outcomes and subsequent costs of patient care.

This systematic literature review summarises the existing literature on hospital‐acquired malnutrition that examines the magnitude of nutritional deterioration amongst adult inpatients and identifies preventable barriers to optimising nutrition support during episodes of care. For the purpose of this review, hospital‐acquired malnutrition is defined as any decline in nutritional status during the course of hospitalisation. The PEO exploratory research question being addressed was: Among adult hospital inpatients (Population), the presence of which modifiable factors (Exposure) contribute to hospital‐acquired malnutrition (Outcome)?

## METHODS

The systematic review protocol was registered with the International Prospective Register of Systematic Reviews (PROSPERO) on 5 July 2020 (CD42020182728). The Preferred Reporting Items for Systematic Review and Meta‐Analyses (PRISMA) guidelines were followed^24^ and a protocol of the review methods was established prior to undertaking the review.

### Search strategy and selection

A pilot search was conducted in PubMed to identify key search terms and to guide the development of the search strategy. Once complete, a literature search was conducted between the 24 April and 30 June 2020. Search terms can be found in the Supporting information (Figure S1). Four electronic databases were searched (CINAHL, MEDLINE, Scopus and PubMed). In addition, all issues of ten top journals in clinical nutrition published during the period from 1 April 2015 to 30 March 2020 were reviewed to ensure that all recent relevant articles were identified (for details, see Supporting information, Figure S2). Search results were exported to EndNote X20 reference management tool (Clarivate Analytics). All titles, abstracts and full‐text articles were screened by a single investigator (AC). A second reviewer (KC) was consulted if there was uncertainty as to whether an article met the inclusion criteria once full‐text articles were retrieved. A narrative synthesis of evidence was completed due to the high level of heterogeneity in methodologies.

### Inclusion and exclusion criteria

Study designs eligible for inclusion were randomised‐controlled trials, cross‐sectional, cohort or case–control studies. This criterion was established to include a broader range of study designs given the more recent shift in research investigations from measuring inpatient malnutrition at a single time point to evaluating patient progression overtime. Consequently, the investigators anticipated drawing from a smaller pool of eligible studies, thus necessitating a broader inclusion criterion to avoid excluding relevant findings. Studies examined adult (≥18 years) male or female inpatients (acute care, sub‐acute or rehabilitation). Included studies measured nutritional status on at least two separate occasions, on admission (or shortly thereafter) and again at a specified time that was sufficiently long to observe a clinically relevant change in nutritional status (>7 days) or just prior to discharge. Only full‐texts available in English were included. Studies examining famine, pre‐existing malnutrition or those conducted in paediatric or pregnant patients, or community‐dwelling, outpatients and nursing home residents were excluded.

### Quality assessment

Using the Academy of Nutrition and Dietetics (AND) Risk of Bias Tool, the methodological quality of each study was assessed by a single investigator (AC) based on the following quality checklist domains: study relevance, research questions, subject selection, group comparability, withdrawals handling, blinding, interventions/exposure, outcomes, analyses, conclusion support and likelihood of bias. A second investigator (KC) was consulted if there was any uncertainty. Studies were assigned a quality rating of positive, negative or neutral. The AND Quality Criteria Checklist was selected as it has been designed to assess the methodological quality for non‐specific research topics within nutrition and dietetics and is applicable across a range of study designs, including cross‐sectional and cohort studies. The Evidence Analysis Manual allows researchers to adapt the assessment to the specific study design by assigning more weight to questions and domains that are specifically relevant to the study design in question.^25^ All relevant articles were assigned a level of evidence based on the National Health and Medical Research Council (NHMRC) criteria, which provides a ranking of the quality of evidence based on the strength and precision of research methods used, the ability to control for bias and to establish cause and effect relationships in humans. Levels of evidence range from highest, Level I, assigned to secondary, preappraised or filtered studies, to lowest, Level IV, assigned to case series, post‐test or post‐test and pretest.^26^

### Data extraction

Key data from selected articles were summarised and tabulated by one reviewer (AC) according to authors, year and country of publication, study design, number of participants, clinical setting, median participant age, nutritional assessment tool and timing, results of nutritional assessment and determinants of malnutrition. *p* values are reported where available.

## RESULTS

### Search results

**Figure 1:**
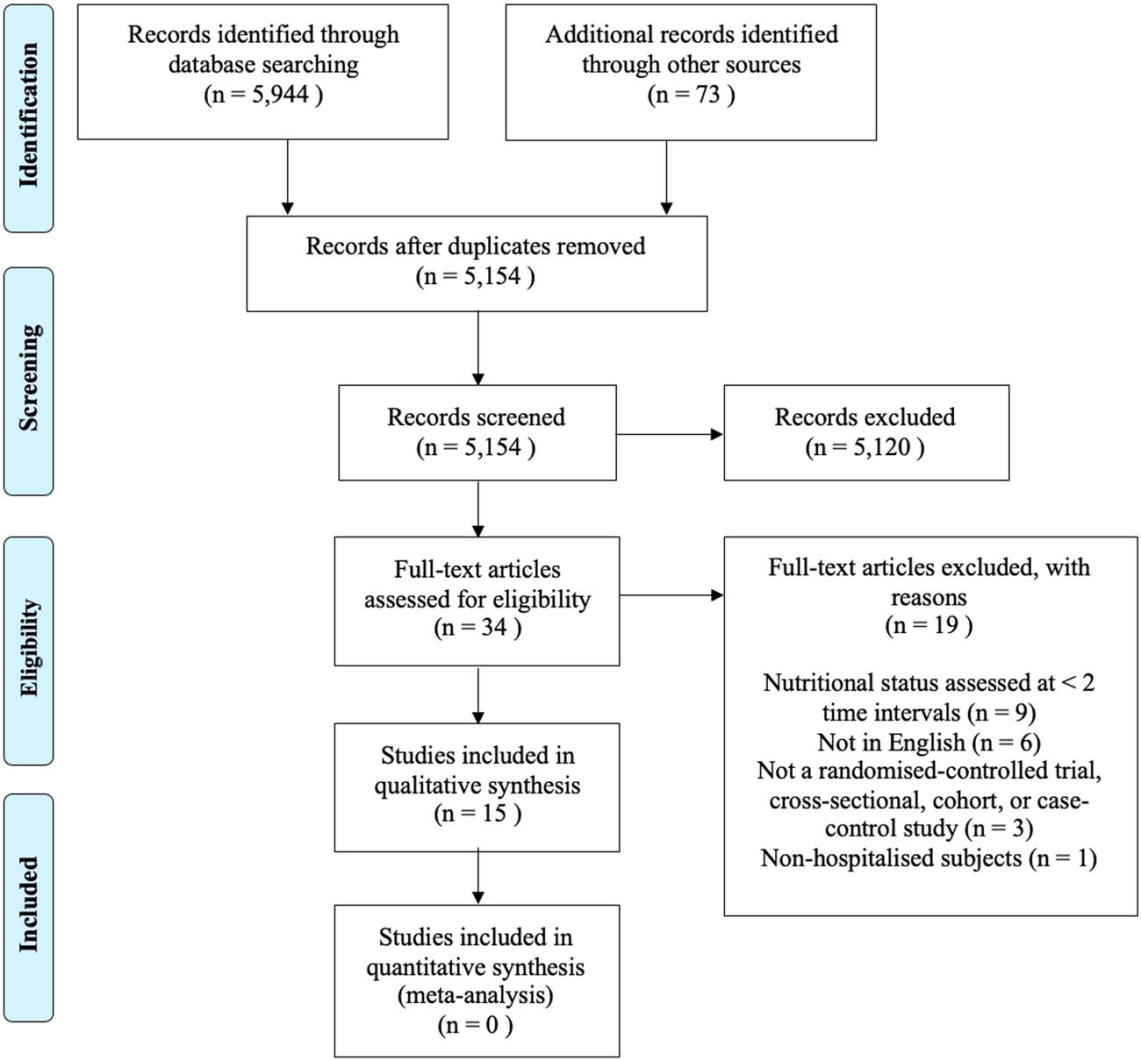
Summary of search and selection process according to the Preferred Reporting Items for Systematic Review and Meta‐Analyses Flowchart (PRISMA).

The search strategy identified 5944 titles from the databases and a further 73 titles from hand‐searching of top journals, resulting in 6017 articles. After 863 duplicates were removed, 5154 titles were screened and 4865 titles were further excluded. Abstracts of 289 articles underwent further screening, leading to retrieval of 34 full‐text articles, of which 15 were eligible for inclusion. A PRISMA flowchart is provided in Figure 1. A full list of full‐text articles that were excluded is available in the Supporting information (Figure S3).

### Study characteristics

Table 1 summarises key characteristics and findings of the included studies. All but one study was observational in design,^27^, ^28^, ^29^, ^30^, ^31^, ^32^, ^33^, ^34^, ^35^, ^36^, ^37^, ^38^, ^39^, ^40^ with the remaining study being quasi experimental.^41^ Eight studies were prospective cohorts,^27^, ^33^, ^34^, ^36^, ^37^, ^38^, ^39^, ^41^ four were prospective cross‐sectional studies,^29^, ^32^, ^35^, ^40^ one was a retrospective cohort study^31^ and one was a sequential explanatory mixed‐methods study.^30^ Four reported on factors associated with deteriorating nutritional status.^28^, ^30^, ^31^, ^36^ Studies were conducted across several countries with representation from both low‐middle and high‐income countries across Africa, Asia, Europe, North America and Oceania.^27^, ^28^, ^29^, ^30^, ^31^, ^32^, ^33^, ^34^, ^35^, ^36^, ^37^, ^38^, ^39^, ^40^, ^41^

**Table 1:** Study characteristics and outcomes

Authors, year (country)	Study design	NHMRC^a^ level of evidence	*n*	Clinical setting	Median age (years)	Assessment tool (intervals)	Prevalence of hospital‐acquired malnutrition	Barriers
Abahuje *et al*. (2020) (Rwanda)^27^	Single‐centre prospective cohort	II^a^	279	Acute care surgery	38	AND/ASPEN^b^ (admission, weekly) SGA^c^ (admission)	Week 1: 41% malnourished. Week 2: 37% malnourished. Week 3: 50% malnourished. Week 4: 43% malnourished.	Not described
Allard *et al*. (2015) (Canada)^28^	Multicentre prospective cohort	II^a^	424	Medical and surgical	68	SGA^c^ (admission, discharge)	19.6% deteriorated, 17.4% improved, 63.0% remained stable.	Dissatisfaction with meal quality and illness‐related effects in medical patients (*p* < 0.01).
Álvarez‐Hernández *et al*. (2012) (Spain)^29^	Multicentre prospective cross‐sectional	IV^a^	1707	Inpatients	63	NRS‐2002^d^ (within 48 h of admission, discharge or day 28 if length of stay > 28 days)	118/1225 (9.6%) well‐nourished on admission became malnourished. 252/351 (72%) malnourished on admission remained malnourished. 99/351 (28.2%) malnourished on admission improved.	Not described
Bell *et al*. (2012) (Australia)^30^	Single‐centre sequential explanatory mixed‐methods	III‐2^a^	44	Orthogeriatric hip fracture patients	81.7	AND/ASPEN^b^ (admission, discharge)	23/44 (52.2%) malnourished at baseline 28/44 (63.6%) malnourished on discharge 9/44 (20.5%) deteriorated	Patient‐perceived: mealtime interruptions (43.1%), poor appetite (36.2%) Clinician‐perceived: Patients do not recognise malnutrition as a problem, low clinical priority amongst clinicians.
Cheng *et al*. (2019) (Australia)^31^	Single‐centre retrospective cohort	III‐2^a^	15419	Inpatients	59	SGA^c^ (undefined)	23/15419 (0.1%) with hospital‐acquired malnutrition 23/419 (5.5%) cases of malnutrition acquired in hospital	Poor appetite (4/23, 25%), meal dissatisfaction (3/23, 19%), operation‐related fasting (3/23, 19%)
Collins *et al*. (2016) (Australia)^32^	Single‐centre prospective cross‐sectional	IV^a^	248	Sub‐acute (rehabilitation, geriatric)	80	MNA^e^ (within 72 h of admission, discharge)	62.0% stable, 10.3% deteriorated, 27.7% improved.	Not described
Diendéré *et al*. (2017) (Burkina Faso)^33^	Single‐centre prospective cohort	II^a^	222	CVA	60.5	BMI < 18.5 kg m^–2^(days 0, 8, 14)	25.2% (95% confidence interval = 19.7–31.5) malnourished at baseline 29.4% (95% confidence interval = 23.2–36.3) malnourished on day 8 31.0% (95% confidence interval = 24.4–38.2) malnourished on day 14	Not described
Haffsteinsdóttir *et al*. (2010) (the Netherlands)^34^	Single‐centre prospective cohort	II^a^	196	Neurology, neurosurgery	68	MNA^e^ (days 0, 10)	Admission: 34% at risk, 7% malnourished, 59% well‐nourished. Day 10: 57% at risk, 22% malnourished, 21% well‐nourished.	Not described
Hosseini *et al*. (2006) (Iran)^35^	Single‐centre prospective cross‐sectional	IV^a^	156	Inpatients	43	BMI^f^ (admission, discharge)	Admission: 9 (5.8%) malnourished, 1 (0.6%) severely malnourished. Discharge: 17 (10.9%) malnourished, 2 severely malnourished (1.3%)	Not described
Incalzi *et al*. (1996) (Italy)^36^	Single‐centre prospective cohort	II^a^	286	General medicine, geriatric	79	MUAC (admission, weekly, discharge)	27% deteriorated	Group with poor intake: rate of poor appetite greater (*p* = 0.001), referred to dietitian no more frequently than those with fair (40%–70%) and good (> 70%) intakes (*p* = 0.49, *p* = 0.76, respectively)
McWhirter *et al*. (1994) (Scotland)^37^	Single‐centre prospective cohort	II^a^	500	General surgery, general medicine, respiratory medicine, orthopaedic surgery, medicine for elderly	Not reported	BMI and TSF or MUAC^g^ (admission, discharge)	16/112 deteriorated.	Not described
Mosselman, *et al*. (2013) (the Netherlands)^38^	Single‐centre prospective cohort	II^a^	73	Acute stroke	65	MNA^e^ (days 0, 10)	15/23 (65%) deteriorated	Not described
Patel *et al*. (2008) (UK)^39^	Single‐centre prospective cohort	II^a^	100	Elderly acute inpatients	82	Demiquet or mindex^h^ (admission, discharge or 4 weeks if length of stay > 4 weeks)	3/100 well‐nourished on admission deteriorated.	Not described
Planas *et al*. (2016) (Spain)^40^	Sub‐analysis of multicentre prospective cross‐sectional	IV^a^	401	Oncology	65	NRS‐2002^d^ (within 48 h of admission, discharge or day 28 if length of stay > 28 days)	Admission: 136/401 (33.9%) malnourished. Discharge: 135/371 (36.4%) malnourished.	Not described
Ramos‐Martínez *et al*. (2016) (Spain)^41^	Single‐centre quasi experimental	III‐2^a^	133	Onco‐haematology	Positive MST at follow‐up (63.4) Negative MST at follow‐up (63.2)	MST^i^ (day 1, weekly)	28/133 (21%) well‐nourished at baseline became malnourished *p < 0.05	Not described

^a^ National Health and Medical Research Council (NHMRC) levels of evidence and grades for recommendations.^42^

^b^ Academy of Nutrition and Dietetics (AND)/American Society for Parenteral and Enteral Nutrition (ASPEN) diagnostic criteria require two of six characteristics to be present for a malnutrition diagnosis (i.e., insufficient energy intake, weight loss, loss of muscle mass, loss of subcutaneous fat, localised or generalised fluid accumulation and diminished functional status based on hand grip strength). Malnutrition is categorised as moderate or severe in the context of acute or chronic illness or injury, or social and environmental circumstances.^43^

^c^ Subjective Global Assessment (SGA) categorises patients as well‐nourished (SGA A), mild‐moderate malnutrition (SGA B) or severe malnutrition (SGA C).^44^

^d^ NRS scores three parameters from 0 to 3. A total score greater than three indicates nutritional risk.^44^

^e^ Mini Nutritional Assessment (MNA) classifies patients at no nutritional risk (12–14 points), at risk of malnutrition (8–11 points) or malnourished (0–7 points).^44^

^f^ Body mass index (BMI) < 18.5 kg m^–2^ (malnourished), BMI < 16.0 kg m^–2^ (severely malnourished).^34^

^g^ BMI < 20 and triceps skinfold (TSF) or mid‐upper arm circumference (MUAC) below 15th centile (mildly undernourished), BMI < 18 and TSF or MUAC below 5th centile (moderately undernourished), BMI < 16 and TSF or MUAC below 5th centile (severely undernourished).^44^

^h^ Demiquet is the weight divided by the square of the demispan in centimetres. Mindex is weight divided by height in metres.^38^

^i^ Malnutrition Screening Tool (MST) classifies nutritional risk based on numerical scores of 0 (low risk), 1 (medium risk) and 2 or higher (high risk).^45^

### Description of assessment methods

All studies evaluated nutritional status or risk on at least two occasions over the course of the admission. This included an assessment or screening on admission as a baseline measure of nutritional status with follow‐ups either at a predetermined time or just prior to discharge. Methods to assess changes in nutritional status were heterogeneous across studies and included the Malnutrition Screening Tool (MST)^41^, ^46^ (*n* = 1), the AND/American Society for Parenteral and Enteral Nutrition (ASPEN) criteria^27^, ^30^ (*n* = 2), the Subjective Global Assessment (SGA)^28^, ^31^ (*n* = 2), the Mini Nutritional Assessment (MNA)^32^, ^34^, ^38^ (*n* = 3) and the Nutrition Risk Screening 2002 tool (NRS‐2002)^29^, ^40^ (*n* = 2), whereas the remaining five studies used either a single or a combination of two anthropometric parameters such as body mass index (BMI), mid‐upper arm circumference (MUAC), triceps skinfolds (TSF) and weight.^33^, ^35^, ^36^, ^37^, ^39^

### Participant characteristics

Mean participant age ranged from 38 to 82 years^27^, ^28^, ^29^, ^30^, ^31^, ^32^, ^33^, ^34^, ^35^, ^36^, ^38^, ^39^, ^40^, ^41^ and one study did not report age.^37^ Clinical specialities varied considerably across studies; 53% (*n* = 8) of studies included patients across two or more clinical areas,^28^, ^29^, ^31^, ^32^, ^35^, ^36^, ^37^, ^39^ and the remaining seven studies recruited only patients from a single clinical specialty or ward.^27^, ^30^, ^33^, ^34^, ^38^, ^40^, ^41^

### Quality assessment

Using the AND Risk of Bias Tool, seven and eight studies were respectively assigned positive and neutral quality ratings, with details of individual studies' quality assessments outlined in the Supporting information (Table S1). According to the NHMRC levels of evidence criteria, eight studies were assessed as Level II evidence, three studies were assessed as Level III‐2 evidence and the remaining four studies were assessed as Level IV evidence.

### Nutritional deterioration

The prevalence of nutritional deterioration was reported to range from 2% to 65% of patients across studies.^27^, ^28^, ^29^, ^30^, ^31^, ^32^, ^33^, ^34^, ^35^, ^36^, ^37^, ^38^, ^39^, ^40^, ^41^ Studies that examined inpatients indiscriminately tended to have lower rates of decline compared to studies that focused on only one or two clinical areas or wards. Nutritional decline amongst more diverse patient cohorts ranged from 5.5% to 20.9%.^29^, ^31^, ^35^, ^37^ Amongst studies that included patients from only one or two clinical areas, sub‐acute rehab and geriatric patients had the most favourable outcomes with 62.0% remaining nutritionally stable, whereas only 10.3% declined, and 27.7% had improved since their baseline assessments.^32^ Neurology and stroke patients experienced the highest rates of nutritional deterioration ranging from 38% to 65% when malnutrition was assessed using a validated screening tool.^34^, ^38^ Notably, on admission, 91% of acute stroke patients were well‐nourished and no patients were malnourished.^38^ When stroke patients were assessed using BMI as an indicator of malnutrition, rates of deterioration dropped to 5.8%.^33^ General medicine and surgery patients showed considerable heterogeneity in rates of deterioration across studies, ranging from 2% to 27%.^27^, ^28^, ^36^ In a Canadian study, 37% of medical and surgical patients experienced a change in nutritional status from admission to discharge; 19.6% of patients across all SGA categories at baseline had deteriorated, whereas 17.0% showed an improvement in nutritional status.^28^ Similarly, 3%–27% of acute geriatric patients were observed to decline.^30^, ^36^, ^39^ Amongst oncology and onco‐haematology patients, 2.5%–21.0%, respectively, deteriorated.^40^, ^41^

### Barriers to nutrition support

Four studies reported on factors associated with decline in nutritional status.^28^, ^30^, ^31^, ^36^ On the institutional level, poor meal quality (taste, appearance and aroma) and satisfaction with the food service was reported by patients from all studies.^28^, ^30^, ^31^, ^36^ Forty‐three per cent of orthogeriatric hip fracture patients reported mealtime interruptions as the most common barrier to intake.^30^ NBM orders and procedure‐related fasting were also frequently reported by Australian inpatients across specialties.^30^, ^31^ Clinicians reported that nutrition being a low clinical priority amongst other healthcare personnel was a major barrier to optimising patient nutrition care.^30^ On the patient level, effects of illness and treatment were consistently reported as barriers across all studies.^28^, ^30^, ^31^, ^36^ Notably, poor appetite was the most common complaint, affecting 25%–55.5% of patients.^30^, ^31^, ^36^ Italian inpatients meeting less than 40% of their prescribed nutritional requirements reported poor appetite significantly more compared to those with greater nutritional intakes.^36^ In Canadian medical patients, loss of appetite was significantly associated with nutritional deterioration (*p* < 0.01).^28^ Additional symptoms reported to inhibit intake included drowsiness, memory, constipation and pain.^28^, ^30^ Chewing and swallowing difficulties were similarly a common complaint affecting intake, particularly in older cohorts.^30^, ^36^ Clinicians perceived that a lack of awareness of malnutrition as a problem by patients was a predominant barrier to meeting nutritional requirements.^30^ When the preventable nature of hospital‐acquired malnutrition was examined, Cheng *et al*.^31^ found that only two of 16 Australian inpatients could be classified as having non‐preventable hospital‐acquired malnutrition because of metabolic derangements that resulted in nutritional needs exceeding the patients' metabolic capacity, despite the provision of adequate nutritional support.

## DISCUSSION

This systematic review characterised the change in nutritional status of hospital inpatients during episodes of care and identified the modifiable determinants associated with nutritional decline. A deeper understanding of the preventable factors associated with hospital‐acquired malnutrition will inform opportunities to guide the development of appropriate preventive strategies and adoption of protocols that target identified institution‐level gaps in practice. Consequently, this may result in both improved patient outcomes and cost‐effectiveness of inpatient care.

This review demonstrated that nutritional compromise is not only an effect of pre‐existing factors, but also generally worsens during inpatient admissions, with hospital‐acquired malnutrition affecting up to 65% of patients, thus supporting the need for repeated nutrition screening and assessment during the course of hospital stay. Barriers to optimising nutritional status at the institutional level were identified by four of the studies reviewed and included poor meal quality and satisfaction, mealtime interruptions, NBM orders and procedure‐related fasting, and low clinical priority amongst both clinical staff and patients. Patient‐level barriers frequently reported included effects of treatment and illness, most notably poor appetite.^28^, ^30^, ^31^, ^36^ In another study, patients who acquired malnutrition during the course of their stay responded positively to oral nutrition supplements, parenteral nutrition (PN) and dietary modifications^41^ which suggests that mitigation is possible with appropriate preventive strategies.

Although all studies reported and compared nutritional status on at least two separate occasions to determine the level and direction of nutritional evolution, only one study reported the true rate of hospital‐acquired malnutrition,^31^ as defined by a deterioration in nutritional status during the course of the admission. As a result of differences in analyses and reporting methods, it is difficult to define the true prevalence of hospital‐acquired malnutrition; however, 2%–65% of patients experienced nutritional deterioration in the included studies.^27^, ^28^, ^29^, ^30^, ^31^, ^32^, ^33^, ^34^, ^35^, ^36^, ^37^, ^38^, ^39^, ^40^, ^41^

All studies demonstrated some level of nutritional deterioration of inpatients over time; however, few studies delineated patients who were malnourished at baseline and improved from those that were previously well‐nourished or malnourished and who saw further declines, likely underestimating the true prevalence of hospital‐acquired malnutrition. Other studies only followed patients who presented as well‐nourished on admission,^31^, ^39^ thus overlooking the two‐thirds of patients already malnourished on admission, who are at risk of deteriorating over the course of their hospitalisation.^4^

Only one study was retrospective in design, which may have contributed to the lower rates of hospital‐acquired malnutrition identified in this study compared to the studies that observed patients prospectively. Because prospective studies reflect ideal research conditions, this may have resulted in more effective identification of hospital‐acquired malnutrition; however it is not necessarily reflective of usual care.^47^

Heterogeneity in the methods used to assess nutritional evolution limited comparability between studies. Many studies could only classify patients into two categories of either well‐nourished or malnourished,^33^, ^35^, ^36^, ^39^ which prevents identification of further nutritional deterioration among already malnourished patients. Moreover, only some studies relied on validated tools to diagnose malnutrition. Tools should be validated for the population in which they are to be used to ensure that they will appropriately detect malnutrition and trigger intervention.^16^ Three studies used BMI^33^, ^35^, ^37^ as a measure of nutritional status, and a fourth study used a modified BMI score.^39^ BMI is often a useful component of more comprehensive nutritional assessments; however, based on current cut‐off points, BMI alone is not adequately sensitive to identify malnourished hospitalised patients.^48^ This is because BMI cannot delineate between body fat and fat‐free mass, particularly relevant to the sarcopaenic population,^49^ nor accommodate changes in fluid resulting from oedema and/or ascites, thereby masking weight loss.^50^ Notably, the lowest rates of deterioration were reported by studies that relied on BMI or demiquet/mindex exclusively as a measure of nutritional status,^33^, ^35^, ^39^ suggesting that BMI or similar measures in isolation may not be adequately sensitive to detect clinically relevant nutritional decline in hospitalised patients, which is consistent with findings from prior research.^47^ Three studies reported on scores using the NRS‐2002 and MST^29^, ^40^, ^46^ nutritional risk screening tools, which are not appropriate for use in diagnosing malnutrition,^48^ whereas the remaining seven studies relied on validated assessment tools such as the AND/ASPEN diagnostic criteria, MNA and SGA.^27^, ^28^, ^30^, ^31^, ^32^, ^34^, ^38^

Future research evaluating the prevalence of hospital‐acquired malnutrition should emphasise accurate assessment of malnutrition using validated assessment tools, rather than screening tools or discrete parameters associated with nutritional status. Furthermore, relying on tools that stratify patients based on the severity of malnutrition provides greater insight into the true rate of hospital‐acquired malnutrition as patients who are already malnourished on admission are not overlooked when clinically relevant deterioration occurs. Although the current review was not limited to include only studies that followed the progression of individual patients from admission to discharge, but rather included studies that compared different cohorts of patients on admission versus on discharge, future research that follows individual patients overtime will enable researchers to delineate patients who were malnourished at baseline and improved, from those who declined during the episode of care to establish a true prevalence of hospital‐acquired malnutrition.

Despite the use of many different criteria to assess hospital‐acquired malnutrition, it commonly occurs globally in countries across Africa, Asia, Europe, North America and Oceania. Although clinical practice guidelines for medical nutrition therapy and monitoring do not differ considerably between nations, disparities in resource availability will impact on the capacity for nutrition care practices to be implemented. For example, patient food services are a standard of Australian hospitals and must adhere to state‐level policies for safety and nutritional adequacy.^14^ At the opposite end of the spectrum, a study of acute surgical patients in Rwanda identified that meals were not provided by the hospital and that patients are expected to be fed by family members or caregivers. PN supplies are also limited as a result of insufficient financial resources in healthcare systems.^27^ Hospital food services and access to nutrition support supplies are an integral component of nutrition care, and these discrepancies in resources are likely to influence the rate of nutritional decline during inpatient admissions. Furthermore, despite greater financial capacities and funding within healthcare systems of developed countries, routine nutritional screening using validated tools is not always performed, and often nutrition intervention is more reactive rather than proactive.^15^, ^16^ A recent study of patients with hospital acquired malnutrition admitted to Australian public hospitals identified that while nutrition screening was routinely undertaken shortly after admission for almost all patients (*n* = 207/208), only one‐third were screened on a weekly basis thereafter. Furthermore, patients with extended lengths of stay were less frequently screened relative to those who had shorter admissions.^51^

Although all studies evaluated patients' nutritional evolution over the course of hospital admissions, only four described barriers to optimising nutritional status.^28^, ^30^, ^31^, ^36^ Furthermore, only one study delineated cases of preventable hospital‐acquired malnutrition that may have been mitigated with timely and appropriate intervention from non‐preventable cases.^31^ In a 2020 retrospective study, Woodward et al found that the odds of developing hospital‐acquired malnutrition increased by 0.6% for each subsequent day of admission; however, whether this is an effect of the hospitalisation or an effect of the illness remains unclear.^47^ Given this gap in our understanding of the specific elements of care that when overlooked or are undervalued have direct repercussions on patients' nutritional status, this presents an important area for future investigations.

Amongst the studies that evaluated barriers to nutritional intake, patients consistently report institutional level barriers including mealtime interruptions, meal dissatisfaction and procedure‐related fasting. Poor appetite, feeling sick and pain on the patient level were common complaints as primary inhibitors to food intake in hospital.^28^, ^30^, ^31^, ^36^ These findings are consistent with those of a recent study in which 85% of patients with hospital‐acquired malnutrition were found to have nutrition impact symptoms and protein and energy intakes less than 80% of prescribed requirements for longer than 2 weeks.^51^ Where oral intake is negatively affected by condition‐ or treatment‐related symptoms, as is often the case in hospitalised patients,^52^ and particularly oncological patients undergoing chemotherapy or radiation therapy, it may be argued that appropriate pharmacological management of symptoms may result in optimised intakes. Lack of provision thereof can be considered to be a modifiable and preventable cause of malnutrition. For example, patients experiencing pain, or nausea and vomiting are often prescribed analgesics and antiemetics, respectively, on an ‘as needed' basis for symptom relief. However, medications charted as such may not be offered unless the patient complains or requests the medication directly. As a result of the busy nature of hospital wards, patients often feel uncomfortable making requests to nursing staff because they fear being a burden and interfering with nurses' abilities to complete other tasks that are perceived to be of higher importance.^53^, ^54^, ^55^

Over 40% of patients in Canadian hospitals reported having been interrupted by staff at mealtimes. When meals were missed, almost 70% of patients were not provided with additional food.^56^ Protected mealtimes have been adopted by some hospitals; however, the results of studies examining the efficacy of these interventions have been inconsistent.^57^, ^58^ Patient meal satisfaction remains low,^56^ although some studies have seen improvements in oral intake with greater attention to the quality and personalisation of the food service. Australian inpatients' energy and protein intake improved significantly with the implementation of a room service foodservice model in which patients order a meal at a time suitable to them, with meal delivery occurring within 45 min.^59^ A bedside menu ordering system similarly showed improvements in patient intake.^60^ Both studies demonstrated reductions in plate waste and food costs.^59^, ^60^ Patient food services directly impact on nutritional status and should prioritise flexibility within the system to better meet patient needs.^15^ The importance of hospital food service quality may not be recognised at times of budget cuts; however, the cost‐savings associated with shorter lengths of stay and reduced rates of complications, which are affected by nutritional status, remain a reminder that overall healthcare costs may be reduced with greater patient meal satisfaction.^1^, ^3^, ^4^, ^6^, ^7^, ^8^, ^9^, ^10^, ^11^, ^12^, ^13^

Where nutritional requirements cannot be met orally despite appropriate mitigation strategies, delays to initiating artificial nutrition support, when indicated, should be avoided to prevent further nutritional deterioration and delayed convalescence.^61^, ^62^, ^63^ Despite it being widely accepted that early initiation of nutrition support remains imperative to preventing nutritional decline, recent findings indicate that more than half of patients with hospital‐acquired malnutrition did not receive nutrition support.^51^ Dietitians consistently report a lack of autonomy concerning the initiation and discontinuation of nutritional support, preventing timely intervention as a result of the resistance met by medical officers and the time spent discussing the appropriateness of such interventions.^15^ However, although this route of feeding promotes improved intake, ethical concerns in relation to artificial nutrition support warrant consideration for the patient's quality of life because oral feeding is an intrinsically social activity. Further, artificial nutrition support may inappropriately prolong death in terminally ill patients and voluntary refusal of nutrition during palliation should be respected.^62^

Two major barriers identified by clinicians were a lack of clinical priority amongst clinicians and limited understanding of malnutrition as a problem amongst patients.^30^ On the patient level, almost all malnourished hip fracture patients failed to recognise their poor nutritional status. Severely inadequate energy and protein intake, in combination with neuropeptide, hormonal and metabolic effects of cachexia, a common physiological feature amongst hip fracture patients, were presumed to contribute to nutritional decline.^30^ At the institutional level, nutrition as a low clinical priority has been identified as a concern for some time, with dietetics personnel reporting that limited autonomy and credibility to perform their respective roles within a multidisciplinary team may contribute to low acknowledgement of nutrition as an important part of medical care amongst non‐dietetics professionals.^15^ This may partly be a consequence of limited provision of formal nutrition education by medical schools.^64^ Although a consensus exists amongst nursing staff regarding the high level of importance of patient nutrition, a lack of clarity regarding nurses' involvement and responsibilities, limited flexibility in food services and the absence of nutrition protocols disincline nursing involvement in nutrition care. Additionally, staffing and time constraints result in competing clinical priorities because treating the acute medical condition is perceived to have a higher level of importance.^65^, ^66^, ^67^, ^68^, ^69^ This makes relying on nursing staff to obtain and record key nutritional information, such as weights and food chart data very challenging. When data used to inform nutrition screening and assessments are unavailable because of staffing constraints, patients are at a greater risk of not being identified for nutritional intervention.^15^ Recio‐Saucedo *et al*.^66^ found that compliance with policies that mandate nutritional screening within 24 h of admission in the UK is positively associated with nurse staffing levels. These findings are consistent with earlier research suggesting that patient care suffers during periods of inadequate staffing.^67^ This association was weakened by higher levels of healthcare assistant staffing which suggests a potential approach to address such staffing challenges by enabling nursing staff to direct their time to other patient‐related activities.^61^ Nurses consistently self‐report that nutrition‐related responsibilities are likely to be neglected when staffing constraints produce competing priorities,^67^ indicating that strategies to address hospital‐acquired malnutrition must be practical when considering the responsibilities of the broader multidisciplinary team rather than nutrition and dietetics personnel only. In the absence of patient information including weights, establishing a malnutrition diagnosis and consequent intervention is likely to be delayed. Obtaining patient weights is well‐documented as a challenge in the clinical setting and when requested by ward dietitians, most patients were not weighed within 24 h. Furthermore, this delay in establishing a diagnosis of malnutrition may prevent timely advocacy for supplemental feeding. This may partly explain why less than one‐third of patients with hospital‐acquired malnutrition had documented recommendations for initiation of nutrition support by dietitians.^51^

Although the preventable nature of hospital‐acquired malnutrition remains unclear, prior research has suggested that up to 95% of hospital‐acquired malnutrition is preventable with appropriate mitigation strategies.^51^ Additional practices have been suggested to address iatrogenic malnutrition through enhanced hospital nutrition care practices. To shift the paradigm of nutrition care, institutions must adopt a culture where nutrition is valued by all members of the multidisciplinary team and administrators, and where all members understand how nutrition care influences patients' broader clinical outcomes and the financial implications of hospital malnutrition.^4^ The Alliance to Advance Patient Nutrition (The Alliance) has made three recommendations to achieve this: (1) educating clinicians on how to recognise and treat malnutrition and discussing this as part of ward rounds; (2) considering malnutrition as part of the patient's medical diagnosis and intervention as a fundamental element of medical care; and (3) cultivating an understanding of the cost savings associated with optimising patient nutrition care amongst administrators, ongoing cost–benefit analyses and revision of budgets to facilitate appropriate preventive strategies.^4^ Furthermore, it is recommended to redefine the roles of members within the multidisciplinary team in the provision of nutrition care. Non‐dietetics professionals from the multidisciplinary team may be assigned a greater level of responsibility in the detection and management of malnutrition by understanding nutrition risk factors and allowing nursing staff autonomy with implementing low risk nutrition care activities.^4^, ^68^, ^69^, ^70^, ^71^ For example, initiating food and fluid charts, previously established enteral nutrition (EN) orders in the interim when awaiting dietetics reviews, and obtaining patient weights when malnutrition is suspected. Allowing dietitians a greater level of autonomy with nutrition care activities such as ordering privileges for therapeutic diets, prescribing oral nutrition supplements or enteral nutrition regimens, and requesting serology can eliminate delays in waiting for physician sign‐off.^4^ Hospitals must implement formal policies and procedures that mandate initial malnutrition screening using tools validated for use by non‐dietetic professionals.^4^ Although this is a requirement in all NSW hospitals, rescreening of patients throughout episodes of care is not required and is often not completed despite it being well‐documented that early identification of malnutrition and timely intervention improves patient outcomes.^14^, ^16^ Given that one‐third of well‐nourished patients are expected to become malnourished, and two‐thirds of patients who present as malnourished on admission are expected to deteriorate,^4^ hospitals seeking to mitigate the consequences of hospital‐acquired malnutrition must establish policies that ensure regular rescreening of patients using simple validated tools, including establishment of individual roles and responsibilities within multidisciplinary teams.^4^, ^16^, ^68^, ^71^ Once the presence of malnutrition is identified, strategies to mitigate further deterioration must be implemented promptly. The Alliance recommends ensuring malnourished or at‐risk patients are fed within 24 h, making every effort to ensure all EN or PN is administered as prescribed, promoting supportive meal environments, flagging when meal consumption is poor, adopting procedures to ensure meal provision when meals are missed, and avoiding NBM orders and holds on EN prior to procedures when practical.^4^ Additionally, clear documentation of nutrition interventions is necessary to enhance communication within the multidisciplinary team, including adopting standardised policies for electronic medical record automatic triggers related to nutritional status. Inclusion of nutrition care plans in discharge summaries enhances continuity of care when patients are transferred to sub‐acute facilities. Further, patients, families and their carers should receive nutrition education and a comprehensive postdischarge care plan which clearly outlines information regarding follow‐up appointments, instructions for nutrition care postdischarge and any recommendations for vitamins, minerals, or oral nutrition supplements.^4^

There are a number of limitations to the findings of this review. First, in some studies, the researchers were unable to follow‐up with all patients and were subsequently excluded from analyses.^27^, ^34^, ^37^, ^38^ This was a result of having been discharged prior to the predetermined follow‐up time intervals or because of death or transfers to other hospitals. However, whether or not the patients that were lost to follow‐up differed significantly in baseline characteristics from those with complete datasets was not reported. Second, because malnutrition has yet to be clearly defined and a gold standard to be established for detecting malnutrition, significant heterogeneity exists between the methods of assessment used in the included studies. Ten of the 15 studies used validated tools,^27^, ^28^, ^29^, ^30^, ^31^, ^32^, ^34^, ^38^, ^40^, ^41^; however, three relied on screening tools.^29^, ^40^, ^41^ The remaining seven studies used three different nutritional assessment tools (MNA, SGA and AND/ASPEN).^27^, ^28^, ^30^, ^31^, ^32^, ^34^, ^38^

Articles retrieved through individual review of journal issues were limited to a 5‐year search period because of the resource‐intensive nature of hand‐searching; thus, it is possible that relevant articles published prior to this search period were not identified. Limiting search periods is a known limitation to systematic reviews because it may omit relevant research. However, hospital‐acquired malnutrition is a relatively new focus in the literature, whereas research conducted prior to this period primarily examined all hospital malnutrition, irrespective of whether acquired prior to or during hospitalisations. Furthermore, utilising a broader study design inclusion criterion contributed to greater heterogeneity amongst the included studies. Heterogeneity of future reviews and the strength of their results may be enhanced by focusing on studies with prospective or retrospective cohort designs. The reliance on a single investigator for conducting the search, screening, extraction and risk of bias assessment is acknowledged as a weakness of this review because there is greater potential for the introduction of systematic and random errors in the absence of double‐screening.

In conclusion, this review highlighted that nutritional deterioration is common among previously well‐nourished and nutritionally compromised patients during hospital admissions. Often, this is a result of preventable barriers to optimal nutrition care that are present at the institutional level. Future research is necessary to determine which strategies are the most effective in preventing or reversing hospital‐acquired malnutrition to optimise patient outcomes. There is a need for institutional nutrition care policies and protocols that outline mandatory monitoring of nutritional status of inpatients at regular intervals to ensure that nutritional risk screening is an ongoing process. Quality improvement initiatives that emphasise patient meal satisfaction are essential to promote optimal intake. Cost–benefit analyses are required to demonstrate the effectiveness of changes to models of care on patient lengths of stay and complications associated with poor nutritional status. Clear establishment of the roles and responsibilities of each member within multidisciplinary healthcare teams in the provision of nutrition care is pivotal to ensuring accountability and mitigating the negative outcomes associated with nutritional decline during hospitalisations.

## AUTHOR CONTRIBUTIONS

Alyssa R. Cass designed the review with support from Karen E. Charlton. Alyssa R. Cass carried out the searches, screened titles, abstracts and full‐text articles against the inclusion criteria in consultation with Karen E. Charlton. Both authors contributed to the interpretation of the results and writing of the manuscript. All authors are in agreement with the manuscript and declare that this review has not been published elsewhere.

## CONFLICTS OF INTEREST

The authors declare that there are no conflicts of interest.

## TRANSPARENCY DECLARATION

The lead author affirms that this manuscript is an honest, accurate and transparent account of the study being reported. The reporting of this work is compliant with PRISMA guidelines. The lead author affirms that no important aspects of the study have been omitted and that any discrepancies from the study as planned (PROSPERO: CD42020182728) have been explained.

## Supporting information

Supporting information.
