# Supplementary material for: Prevalence of hospital‐acquired malnutrition and modifiable determinants of nutritional deterioration during inpatient admissions: A systematic review of the evidence
Source: J Hum Nutr Diet. 2022 Apr 26;35(6):1043–58. doi: 10.1111/jhn.13009 (PMC9790482; doi:10.1111/jhn.13009)
Supplement: Supplementary file 1 — Supporting information. [file JHN-35-1043-s001.docx]

**Supplementary figure 1: Search terms**

1. Hospital-acquired malnutrition
2. In-hospital malnutrition
3. Nosocomial malnutrition
4. Protein-energy malnutrition
5. Delivery of health care
6. Gaps in care
7. Health knowledge, attitudes, practice
8. Nutrition care
9. Nutrition therapy
10. Nutritional status
11. Nutritional surveillance
12. Quality of health care
13. Quality improvement
14. Academic medical centres
15. Hospitalization
16. Hospitals
17. Inpatients
18. Tertiary healthcare
19. Prevalence
20. Risk factors
21. 1 OR 2 OR 3 OR 4
22. 5 OR 6 OR 7 OR 8 OR 9 OR 10 OR 11 OR 12 OR 13
23. 14 OR 15 OR 16 OR 17 OR 18
24. 19 OR 20
25. 21 AND 22 AND 23 AND 24
26. Child
27. Paediatric
28. Pregnancy
29. Eating disorder
30. Famine
31. Vitamin
32. Community
33. 26 OR 27 OR 28
34. 29 OR 30 OR 31
35. 32 AND 33 AND 34
36. 25 NOT 35

**Supplementary figure 2: Hand-searched journals**

1. American Journal of Clinical Nutrition
2. British Journal of Nutrition
3. Clinical Nutrition ESPEN
4. European Journal of Clinical Nutrition
5. Journal of the Academy of Nutrition and Dietetics
6. Journal of Human Nutrition and Dietetics
7. Journal of Parenteral and Enteral Nutrition
8. Nutrition & Dietetics
9. Nutrition in Clinical Practice
10. Nutrition Journal

**Supplementary figure 3: List of excluded articles**

1. Keller H, Allard J, Vesnaver M et al. (2015) Barriers to food intake in acute care hospitals: a report of the Canadian Malnutrition Task Force. J Hum Nutr Diet 28, 546-57.
2. Graeb F, Wientjens R, Wolke R et al. (2020) Changes in nutritional status of geriatric patients during hospital treatment. Aktuel Ernahrunsmed 45, 16-24.
3. Halvorsen K, Eide HK, Sortland K et al. (2016) Documentation and communication of nutritional care for elderly hospitalized patients: Perspectives of nurses and undergraduate nurses in hospitals and nursing homes. BMC Nurs 15, 70.
4. Chambers R, Bryan J, Jannat-Khah et al. (2019) Evaluating gaps in care of malnourished patients on general medicine floors in an acute care setting. Nutr Clin Pract 34, 313-318.
5. Byrnes A, Young A, Mudge A et al. (2019) Exploring practice gaps to improve PERIoperativE Nutrition CarE (EXPERIENCE Study): a qualitative analysis of barriers to implementation of evidence-based practice guidelines. Eur J Clin Nutr 73, 94-101.
6. Simzari K, Vahabzadeh D, Nouri Saeidlou S et al. (2017) Food intake, plate waste and its association with malnutrition in hospitalized patients. Nutr Hosp 34, 1376-81.
7. Hiesmayr M, Tarantino S, Moick S et al. (2019) Hospital malnutrition, a call for political action: a public health and NutritionDay perspective. J Clin Med 8, 2048.
8. Holmes S (1996) The incidence of malnutrition in hospitalised patients. Nurs Times 92, 43-5.
9. Pinchcofsky GD, Kaminski MV (1985) Increasing malnutrition during hospitalization: documentation by a nutritional screening program. J Am Coll Nutr 4, 471-9.
10. Lorton CM, Griffin O, Higgins K et al. (2020) Late referral of cancer patients with malnutrition to dietitians: a prospective study of clinical practice. Support Care Cancer 28, 2351-60.
11. Pérez de la Cruz A, Lobo Támer G, Orduña Espinosa C et al. (2004) Malnutrition in hospitalized patients: prevalence and economic impact. Med Clin (Barc) 123, 201-6.
12. Antonelli Incalzi R, Pagano F, Bruno E et al. (1995) Malnutrition in the acute care hospital: a very common problem. Ann Ital Med Int 10, 222-6.
13. Rinninella E, Cintoni M, De Lorenzo A et al. (2019) May nutritional status worsen during hospital stay? A sub-group analysis from a cross-sectional study. Intern Emerg Med 14, 51-7.
14. Duerksen DR, Keller HH, Vesnaver E et al. (2016) Nurses’ perceptions regarding the prevalence, detection, and causes of malnutrition in Canadian hospitals. JPEN J Parenter Enteral Nutr 40, 100-106.
15. Dzieniszewski J, Jarosz M, Szczygiel J et al. (2003) Nutrition state of patients in hospitals in Poland I. Screening assessment of older patients. Pol Merkur Lekarski 15, 144-150.
16. Paillaud E, Campillo B, Bories PN et al. (2001) Nutritional status in 57 elderly patients hospitalized in a rehabilitation unit: Influence of causing disease. Rev Méd Interne 22, 238-244.
17. Fernández López MT, Fidalgo Baamil O, López Doldán C et al. (2014) Prevalence of malnutrition in not critically ill inpatients. Nutr Hosp 30, 1375-83.
18. Kingston P, Donaldson-Smith J, McDougall T (2017) Prevention and treatment of hospital acquired malnutrition through nutritional screening and nutrition care planning. Clin Nutr ESPEN 22, 128-9.
19. McFadden C (2019) RDN Audits to determines the prevalence of hospital malnutrition reveal a need to shift the focus of quality improvement efforts. J Acad Nutr Diet 119, S59.

Supplementary table 1: Quality rating using the Academy of Nutrition and Dietetics Risk of Bias Tool

| Criteria | Abahuje E et al^24^ | Allard JP et al^25^ | Álvarez-Hernández J et al^26^ | Bell J et al^27^ | Cheng J et al^28^ | Collins J et al^29^ | Diendéré J et al^30^ | Hafsteinsdóttir TB et al^31^ |
| --- | --- | --- | --- | --- | --- | --- | --- | --- |
| Relevance | + | + | + | + | + | + | + | + |
| Was the research question clearly stated? | Yes | Yes | Yes | Yes | Yes | Yes | Yes | Yes |
| Was the selection of study subjects/patients free from bias? | Yes | Yes | Yes | Yes | Yes | Yes | No | Unclear |
| Were study groups comparable? | N/A | N/A | N/A | Yes | N/A | N/A | N/A | N/A |
| Was method of handling withdrawals described? | Yes | Yes | Yes | Yes | Yes | Yes | Yes | Yes |
| Was blinding used to prevent introduction of bias? | No | No | No | No | No | No | No | No |
| Were intervention/therapeutic regiments/exposure factor or procedure and any comparison(s) described in detail? Were intervening factors described? | Yes | Yes | Yes | Yes | Yes | Yes | Unclear | Yes |
| Were outcomes clearly defined and the measurements valid and reliable? | Yes | Yes | Yes | Yes | Yes | Yes | Unclear | Yes |
| Was the statistical analysis appropriate for the study design and type of outcome indicators? | Yes | Yes | Yes | Unclear | Yes | Yes | Yes | Yes |
| Are conclusions supported by results with biases and limitations taken into consideration? | Yes | Yes | Yes | Yes | Yes | Yes | No | Yes |
| Is bias due to study’s funding or sponsorship unlikely? | Yes | Yes | Yes | Unclear | Yes | Yes | Yes | Unclear |
| Quality Rating | + | + | + | + | + | + | ∅ | ∅ |

| Criteria | Hosseini S et al^32^ | Incalzi RA et al^33^ | McWhirter JP & Pennington CR^34^ | Mosselman MJ et al^35^ | Patel MD & Martin FC^36^ | Planas M et al^37^ | Ramos-Martínez T et al^38^ |
| --- | --- | --- | --- | --- | --- | --- | --- |
| Relevance | + | + | + | + | + | + | + |
| Was the research question clearly stated? | Yes | Yes | Yes | Yes | Yes | Yes | Yes |
| Was the selection of study subjects/patients free from bias? | Yes | Yes | Yes | Yes | Yes | Yes | Yes |
| Were study groups comparable? | N/A | N/A | N/A | N/A | N/A | N/A | N/A |
| Was method of handling withdrawals described? | No | Unclear | No | Yes | Yes | No | No |
| Was blinding used to prevent introduction of bias? | Yes | No | Unclear | No | Yes | No | No |
| Were intervention/therapeutic regiments/exposure factor or procedure and any comparison(s) described in detail? Were intervening factors described? | Yes | Yes | Yes | Unclear | Unclear | Yes | Yes |
| Were outcomes clearly defined and the measurements valid and reliable? | No | No | No | Yes | No | Unclear | Yes |
| Was the statistical analysis appropriate for the study design and type of outcome indicators? | Unclear | No | Unclear | Yes | Unclear | Yes | Unclear |
| Are conclusions supported by results with biases and limitations taken into consideration? | No | Yes | Unclear | Yes | Yes | Yes | Yes |
| Is bias due to study’s funding or sponsorship unlikely? | Unclear | No | Unclear | Yes | Unclear | Yes | Unclear |
| Quality Rating | ∅ | ∅ | ∅ | ∅ | ∅ | ∅ | + |
